# Increase of Neutrophil Extracellular Traps, Mitochondrial DNA and Nuclear DNA in Newly Diagnosed Type 1 Diabetes Children but Not in High-Risk Children

**DOI:** 10.3389/fimmu.2021.628564

**Published:** 2021-06-15

**Authors:** Camilla Skoglund, Daniel Appelgren, Ingela Johansson, Rosaura Casas, Johnny Ludvigsson

**Affiliations:** ^1^ Department of Biomedical and Clinical Sciences, Linköping University, Linköping, Sweden; ^2^ Department of Health, Medicine and Caring Sciences, Linköping University, Linköping, Sweden; ^3^ Crown Princess Victoria Children’s Hospital, Region Östergötland, Linköping, Sweden

**Keywords:** type 1 diabetes (T1D), T1D high-risk, neutrophil extracellular traps (NETs), mitochondrial DNA (mtDNA), nuclear DNA (nDNA), inflammation

## Abstract

Neutrophil extracellular traps (NETs) and mitochondrial DNA (mtDNA) are inflammatory mediators involved in the development of type 1 diabetes (T1D). Pancreas-infiltrating neutrophils can release NETs, contributing to the inflammatory process. Levels of NETs are increased in serum from patients with T1D and mtDNA is increased in adult T1D patients. Our aim was to investigate extracellular DNA (NETs, mtDNA and nuclear DNA) in children with newly diagnosed T1D and in children at high risk of the disease. We also elucidated if extracellular DNA short after diagnosis could predict loss of endogenous insulin production. Samples were analysed for mtDNA and nuclear DNA using droplet digital PCR and NETs were assessed by a NET-remnants ELISA. In addition, *in vitro* assays for induction and degradation of NETs, as well as analyses of neutrophil elastase, HLA genotypes, levels of c-peptide, IL-1beta, IFN and autoantibodies (GADA, IA-2A, IAA and ZnT8A) were performed. In serum from children 10 days after T1D onset there was an increase in NETs (p=0.007), mtDNA (p<0.001) and nuclear DNA (p<0.001) compared to healthy children. The elevated levels were found only in younger children. In addition, mtDNA increased in consecutive samples short after onset (p=0.017). However, levels of extracellular DNA short after onset did not reflect future loss of endogenous insulin production. T1D serum induced NETs *in vitro* and did not deviate in the ability to degrade NETs. HLA genotypes and autoantibodies, except for ZnT8A, were not associated with extracellular DNA in T1D children. Serum from children with high risk of T1D showed fluctuating levels of extracellular DNA, sometimes increased compared to healthy children. Therefore, extracellular DNA in serum from autoantibody positive high-risk children does not seem to be a suitable biomarker candidate for prediction of T1D. In conclusion, we found increased levels of extracellular DNA in children with newly diagnosed T1D, which might be explained by an ongoing systemic inflammation.

## Introduction

Neutrophils and their inflammatory mediators have been shown to be involved in various autoimmune diseases including diabetes (1, 2). The number of neutrophils in blood is reduced in patients with type 1 diabetes (T1D) and in pre-symptomatic autoantibody positive at-risk individuals (3–5). Pancreas infiltration of neutrophils both before symptoms and at disease onset can explain the reduced number in blood (5). It has also been shown that some of these pancreas-infiltrating neutrophils release neutrophil extracellular traps (NETs) (5). NETs, which appear as web-like structures, are released during a cell death process called NETosis and contain cytosolic and granule proteins as well as DNA, often both of nuclear (nDNA) and mitochondrial (mtDNA) origin (6–8). Neutrophils can also actively release mtDNA without parallel release of nDNA during so called vital NETosis (9) or in the form of interferogenic mitochondrial DNA webs (10). NETs and other extracellular DNA are degraded by DNases but if not properly cleared or if increased release, or both, the prolonged exposure of these structures to the immune system can lead to inflammation (11). Histones, mtDNA and many of the proteins in NETs are inflammatory (7, 12). Other inflammatory mediators released by neutrophils upon activation include degranulation of lytic enzymes, reactive oxygen species (ROS) and cytokines (13). Increased levels of these toxic substances might lead to destruction of the beta cells. Higher levels of inflammatory cytokines in newly diagnosed T1D patients (14–16) and in high-risk children (17) suggest activation of a systemic inflammation, also early in the T1D process, and might be a sign of ongoing beta cell destruction. In line with this, TNF and IL-1beta have been shown to induce NETosis (18). Increased NET formation has been found in both type 1 and type 2 diabetes patients (19–23). Neutrophils from T1D patients are more prone to produce NETs (19) and recently also the composition of NETs was shown to be different in T1D (24).

Increased levels of mtDNA in serum from adult T1D patients has been observed (25) as well as an increase of NETs in serum from children with T1D, most pronounced in children with a disease duration of less than one year (4). Circulating levels of NETs as well as mtDNA and nDNA in children at high risk of T1D have not been explored so far. Based on the findings above we hypothesized that increased levels of NETs in children with T1D could be accompanied by increased levels of mtDNA and nDNA, and that also autoantibody positive children at high risk of T1D have increased levels of NETs as well as extracellular mtDNA and nDNA, all of these collectively referred to as extracellular DNA in this article. We therefore measured levels of extracellular DNA in children short after onset of T1D, and in children positive for multiple islet autoantibodies considered to be at high risk of T1D, and compared with matched healthy controls. We also explored whether extracellular DNA in serum short after diagnosis could predict loss of endogenous insulin production.

## Material and Methods

### Study Populations

In total, three cohorts of children have been included; one cohort to study differences between T1D children very short after diagnosis and healthy controls (cohort 1), one to study longitudinal changes in newly diagnosed T1D children (cohort 2) and one to study high-risk children longitudinally and to compare with healthy controls (cohort 3, **Supplementary Figure 1**, **Table 1**). At the paediatric clinic, Linköping University Hospital, blood samples are routinely collected from T1D children 10 days, 1 month and 3 months after diagnosis. Cohort 1 consists of 50 randomly selected newly diagnosed T1D children with samples collected at 10 days after diagnosis, 25 boys and 25 girls, with a median age of 8.4 years (IQR 6.2-10.8), and 42 age matched healthy controls with a median age of 9.8 years (IQR 9.5-16). From 20 of the T1D children additional blood samples taken at 1 and 3 months after diagnosis were included. Cohort 2 consists of 12 newly diagnosed T1D children, 7 boys and 5 girls, with a median age of 14.5 years (IQR 14.0-15.8). They had participated in an intervention study in which they received placebo (26). Consecutive samples were taken at 2, 3, 4, 5, 8, 17 and 32 months after onset. Cohort 3 consists of 20 children at high risk of T1D, 13 boys and 7 girls, with a median age of 10.6 years (IQR 10.2-11.4), and 17 age and sex matched healthy controls, 11 boys and 6 girls, with a median age of 12 years (IQR 11.6-12.3). Both the high-risk children and the healthy controls were recruited from the prospective ABIS (All Babies in Southeast Sweden) study representing the general Swedish population. The high-risk children were positive for multiple islet autoantibodies and at the age of 10-12 years they were enrolled in a follow-up study of high-risk individuals previously described (27, 28). From the high-risk children blood was collected 5 times with 6 months interval. One child was excluded due to sample haemolysis. Eleven of the high-risk children developed T1D during or short after the follow-up study (progressors).

Table 1Characteristics of the three cohorts of children included in the study.

**Table:** 

Cohort 1 - Type 1 diabetes children and healthy controls						
	Type 1 diabetes	Type 1 diabetes - paired samples			Healthy controls	p-value
	10 days	10 days*	1 month	3 months		
Individuals [number, (male/female)]	50 (25/25)	20 (10/10)	20 (10/10)	20 (10/10)	42 (na/na)	
Median age [years, (IQR)]	8.4 (6.2-10.8)	6.9 (5.6-9.1)			9.8 (9.5-16)	
GADA [units/ml, (IQR)]	81 (14-297) (10 mv)	126 (22-375) (6 mv)			15 (11-26) (1 mv)	
IA2A [units/ml, (IQR)]	175 (6-250) (10 mv)	250 (125-250) (6 mv)			2.7 (2.7-2.7)	
IAA [units/ml, (IQR)]	4.2 (0.1-16.1) (3 mv)	5.3 (0.7-15.3)			na	
ZnT8A RA (Arg) [units/ml, (IQR)]	119 (10-800) (39 mv)	139 (24-653) (16 mv)			na	
ZnT8A WA (Trp) [units/ml, (IQR)]	42 (15-129) (39 mv)	37 (15-111) (16 mv)			na	
ZnT8A QA (Glt) [units/ml, (IQR)]	37 (23-320) (39 mv)	30 (20-47) (16 mv)			na	
Autoantibody negative**[%, (number)]	14 (4) (21 mv)	7 (1) (6 mv)			100 (42)	
Positive for 1 autoantibody** [%, (number)]	24 (7) (21 mv)	7 (1) (6 mv)			0 (0)	
Positive for 2 autoantibodies**[%, (number)]	21 (6) (21 mv)	43 (6) (6 mv)			0 (0)	
Positive for 3 autoantibodies** [%, (number)]	41 (12) (21 mv)	50 (7) (6 mv)			na	
HLA-DQ risk High/Moderate/Low [number]	11/20/7 (12 mv)	4/8/1 (7 mv)			0/5/22 (2 mv)	
C-peptide [nmol/l, (IQR)]	0.10 (0.05-0.15) (5 mv)	0.06 (0.02-0.10) (5 mv)	0.14 (0.06-0.24) (5 mv)	0.14 (0.08-0.26) (5 mv)	0.53 (0.31-0.93)	<0.001 ¤, 0.021 #
HbA1c [mmol/mol, (IQR)]	110 (81-147) (10 mv)	97 (79-121) (3 mv)	59 (52-63) (10 mv)	42 (38-49) (4 mv)	na	<0.001 #
Fasting glucose [mmol/l, (IQR)]	7.0 (5.1-16.4) (22 mv)	11.1 (6.6-25.8) (9 mv)	na	5.8 (4.7-7.6) (4 mv)	na	0.008 #
120 min OGTT [mmol/l, (IQR)]	na	na	na	17.3 (13.2-21.9) (4 mv)	na	

^*^subgroup of all type 1 diabetes samples at 10 days after diagnosis. ^**^GADA, IA2A and IAA included for diabetes group, GADA and IA-2A for healthy controls. ^¤^Type 1 diabetes 10 days v.s. healthy controls. ^#^Paired samples. na, not available. mv, missing values.

**Table:** 

Cohort 2 - Type 1 diabetes children								
	2 months	3 months	4 months	5 months	8 months	17 months	32 months	p-value
Individuals [number, (male/female)]	12 (7/5)							
Median age at onset [years, (IQR)]	14.5 (14.0-15.8)							
Time after onset [months, (IQR)]	2.3 (2.0-3.6)							
HLA-DQ risk High/Moderate/Low [number]	3/6/1 (2 mv)							
GADA [units/ml, (IQR)]	215 (112-907)	194 (120-1095)	195 (121-967)	189 (116-802)	138 (103-865)	163 (100-792)	94 (51-314)	
IA2A [units/ml, (IQR)]	63 (3-1166)	na	na	na	141 (3-750)	na	na	
Fasting c-peptide [nmol/l, (IQR)]	0.20 (0.13-0.29)	0.26 (0.14-0.32)	0.22 (0.18-0.30)	0.25 (0.16-0.44)	0.21 (0.15-0.28)	0.17 (0.06-0.32)	0.08 (0.05-0.14)	<0.001
C-peptide [AUC, (IQR)]	0.72 (0.46-0.83)	na	na	na	0.70 (0.43-0.85)	0.39 (0.25-0.63)	0.22 (0.13-0.35)	<0.001
HbA1c [mmol/mol, (IQR)]	42.5 (38.5-50.3)	39.0 (36.5-45.0)	36.5 (33.3-43.3)	38.5 (33.5-43.5)	39.0 (35.8-50.0)	51.0 (39.8-53.8)	55.5 (46.0-68.5)	<0.001

AUC, area under curve. na, not available. mv, missing values.

**Table:** 

Cohort 3 - Children with high risk of type 1 diabetes and healthy controls							
	High-risk children					Healthy controls	p-value
	Baseline	6 months	12 months	18 months	24 months		
Individuals [number, (male/female)]	20 (13/7)					17 (11/6)	
Progressors [number, (%)]	11 (55)						
Median age [years, (IQR)]	10.6 (10.2-11.4)					12.0 (11.6-12.3)	
Weight [kg, (IQR)]	40 (35-45) (1 mv)	na	45 (39-51) (2 mv)	59 (41-75) (16 mv)	55 (53-69) (12 mv)	43 (36-50)	
GADA [units/ml, (IQR)]	567 (146-5935)	440 (88-4950) (1 mv)	420 (69-4713) (3 mv)	483 (59-3578) (11 mv)	159 (105-2966) (9 mv)	10 (7-15)	
IA2A [units/ml, (IQR)]	138 (3-420)	14 (3-493) (1 mv)	9 (3-607) (3 mv)	172 (3-1210) (11 mv)	228 (3-1321) (9 mv)	2.7 (2.7-2.7)	
IAA [units/ml, (IQR)]	6.7 (3.8-12.9)	5.4 (0.1-13.9) (1 mv)	7.2 (3.9-15.1) (3 mv)	12.6 (7.0-28.8) (11 mv)	15.7 (9.6-37.7) (9 mv)	0.1 (0.1-0.1) (1mv)	
ZnT8A RA (Arg) [units/ml, (IQR)]	217 (5-1045)	224 (5-821) (1 mv)	180 (5-823) (3 mv)	243 (83-1153) (11 mv)	254 (5-1525) (9 mv)	5 (5-5) (1mv)	
ZnT8A WA (Trp) [units/ml, (IQR)]	5 (5-127)	5 (5-66) (1 mv)	10 (5-148) (3 mv)	30 (5-458) (11 mv)	19 (5-422) (9 mv)	16 (6-19) (1mv)	
ZnT8A QA (Glt) [units/ml, (IQR)]	5 (5-44)	5 (5-18) (1 mv)	5 (5-22) (3 mv)	15 (5-201) (11 mv)	5 (5-115) (9 mv)	5 (5-5) (1mv)	
Autoantibody negative** [number]	0	0 (1 mv)	0 (3 mv)	0 (11 mv)	0 (9 mv)	16 (1mv)	
Positive for 1 autoantibody** [number]	0	0 (1 mv)	0 (3 mv)	0 (11 mv)	1 (9 mv)	0 (1 mv)	
Positive for 2 autoantibodies** [number]	8	11 (1 mv)	10 (3 mv)	3 (11 mv)	3 (9 mv)	0 (1 mv)	
Positive for 3 autoantibodies** [number]	7	4 (1 mv)	2 (3 mv)	2 (11 mv)	3 (9 mv)	0 (1 mv)	
Positive for 4 autoantibodies** [number]	5	4 (1 mv)	5 (3 mv)	4 (11 mv)	4 (9 mv)	0 (1 mv)	
HLA-DQ risk High/Moderate/Low [number]	4/9/7					1/1/14	
Fasting C-peptide [nmol/l, (IQR)]	0.34 (0.31-0.42)	0.39 (0.34-0.49) (1 mv)	0.39 (0.30-0.52) (3 mv)	0.44 (0.31-0.65) (11 mv)	0.43 (0.39-0.80) (9 mv)	0.47 (0.36-0.57)	0.001 #, 0.002 ¤
HbA1c [mmol/l, (IQR)]	35.0 (33.0-36.0)	35.0 (33.0-37.0) (1 mv)	36.0 (34.0-36.5) (3 mv)	34.0 (32.5-40.5) (11 mv)	38.0 (35.0-41.0) (9 mv)	na	0.003 #
Fasting glucose [mmol/l, (IQR)]	5.3 (5.1-5.7)	5.5 (5.2-5.7) (2 mv)	5.4 (5.0-5.8) (2 mv)	5.4 (5.1-5.7) (11 mv)	5.4 (5.2-5.8) (9 mv)	5.1 (4.9-5.3) (1 mv)	ns #, ns ¤
120 min OGTT [mmol/l, (IQR)]	6.9 (6.1-8.9)	na	7.5 (6.8-8.3) (4 mv)	na	6.4 (6.1-9.1) (13 mv)	na	ns #

na, not available. ^¤^High risk v.s. healthy controls. ^#^Within high-risk group. mv, missing values. ns, non-significant.

All patients and their parents, as well as healthy individuals and their parents, had given their informed consent to participate in these studies of development of T1D. The Research Ethics Committee of the Faculty of Medicine and Health Sciences at Linköping University, the Medical Faculty at Lund University and the Medical Product Agency in Sweden have given ethical approval for the study.

### Methods

#### Blood Sampling and Oral Glucose Tolerance Test

Samples were drawn after fasting overnight, except for healthy controls in cohort 1. Oral glucose tolerance test (OGTT) was performed as described previously (27). Samples from cohort 1 were stored at -20°C and samples from cohort 2 and 3 were stored at -80°C (**Supplementary Figure 1**). The main reason for having two different healthy control groups, one in cohort 1 and one in cohort 3, was to avoid comparison of samples stored at different temperatures.

#### NET-Remnants Assay

A NET-remnants ELISA, detecting complexes of DNA and myeloperoxidase (MPO) both found in NETs, was run for all samples (cohort 1-3) as previously described (29). NET-remnants will be referred to as NETs for the rest of the article. Briefly, the plate was coated at 4°C overnight with anti-MPO (DAKO, Carpinteria, CA, USA) followed by washing (0,9% NaCl and 0,05% Tween 20 in deionized H_2_O) and addition of a peroxidase-labelled anti-DNA antibody (detection antibody of the Human Cell Death Detection ELISAPLUS; Roche Diagnostics GmbH, Mannheim, Germany) together with serum sample. After 2 hours of incubation the plate was washed, ABTS-substrate (Roche Diagnostics) added and after 40 min at 37°C the plate was read at 405 nm in a VersaMax ELISA microplate reader (Molecular Devices, Sunnyvale, CA, USA). All samples were run in duplicates, a 9-point standard curve with NETs produced in the lab (29) was included on each plate as well as positive control and blank. Values were interpolated from the standard curve and expressed as arbitrary units. Samples below detection limit were given the value 0.

#### Neutrophil Elastase Assay

Neutrophil elastase was measured with Human Neutrophil Elastase SimpleStep ELISA kit (Abcam) according to the manufacturer’s instructions. Samples were run in duplicates and results were presented as OD values at 405 nm.

#### Visualization of NETs

The experiment was performed according to a previously published protocol (30) with some modifications. Briefly, neutrophils were isolated from an adult healthy individual and seeded onto poly-L-lysine (0.001%, Sigma Aldrich) coated coverslips at a density of 200 000 cells per well in 24-well plates. The ability of T1D serum to induce NETs was assessed by addition of 5% serum, from three T1D patients at 10 days and at 3 months after disease onset, to the neutrophils and incubated for 4 hours at 37°C in 5% CO2. Phorbol-12-myristate-13-acetate (PMA) at a concentration of 20 nM was used as a positive control for NET generation and medium as a negative control. To investigate the ability of T1D serum to degrade NETs, PMA-induced NETs were incubated with 1% of serum from three T1D patients at 10 days after disease onset and from three healthy controls, for 16 hours. DNase-I (4 U/ml) was used to show complete degradation of NETs and medium to show no or little degradation of NETs. After incubation, the cells were fixed with 4% formaldehyde solution and unspecific binding was blocked with 2% bovine serum albumin (BSA). Between each step the coverslips were washed with PBS. A primary antibody (monoclonal rabbit anti-human NE, ab131260, Abcam, 1:70) diluted in 2% BSA was added and incubated for 1 hour followed by incubation with a secondary antibody (polyclonal goat anti-rabbit IgG conjugated with Alexa Fluor 488, ab150077, Abcam, 1:500) also for 1 hour. Finally, the coverslips were mounted onto slides with ProLong Gold Antifade Reagent with DAPI, and after 24 h, in the dark, the slides were examined with an immunofluorescence microscopy (Leica DMi8, 25x objective).

#### Quantification of NETs

Neutrophils from an adult healthy individual were seeded at a density of 50 000 cells/well in black 96-well immuno plates (Thermo Fisher) coated with poly-L-lysine and allowed to settle for 1h at 37°C in 5% CO2. For the NET-induction experiment, Sytox Green (Invitrogen) was added at a concentration of 2.5 µM and after 15 minutes the fluorescence was measured using a GloMax plate reader (GloMax Explorer version 3.1.0, Promega, excitation 475 nm and emission 500-550 nm). Thereafter, the cells were stimulated with PMA (final concentration 20 nM) or 5% serum from three T1D patients at 10 days and at 3 months after disease onset. Triton X-100 (1%, Sigma-Aldrich) and medium were used as controls. All samples were run in duplicates. After 4h at 37°C in 5% CO2 the fluorescence was again measured with GloMax, and for each well the fluorescence from the initial measurement was subtracted. For the NET-degradation experiment, cells were seeded as explained above and NETs were induced by stimulation with PMA (final concentration 20 nM) for 4h at 37°C in 5% CO2. Thereafter, the supernatant was carefully removed and 1% serum from three T1D patients at 10 days after disease onset and from three healthy controls, diluted in DNase buffer (10 mM Tris-HCl, pH 7.5, 50 mM NaCl, 10 mM MgCl2 and 2 mM CaCl2), were added to the wells. DNase-I (20 U/ml), Triton X-100 (1%) and DNase buffer alone were used as controls. After 16 hours of incubation at 37°C in 5% CO2 aliquots of the supernatants were transferred to a new plate and EDTA was added to stop the DNase activity (final concentration 2 mM). Pico Green was added to the samples, according to the instructions in the Quant-IT PicoGreen dsDNA Assay Kit (Invitrogen), and the fluorescence was measured with the GloMax reader. Serum at 1% diluted in DNase buffer was incubated without neutrophils in a 96-well plate for 16h at 37°C in 5% CO2 to measure the initial DNA concentrations in the serum from the six individuals. This DNA concentration was subtracted from the values obtained in the final fluorescence measurement.

#### DNA Extraction and Droplet Digital PCR (ddPCR) for Detection of mtNDA and nDNA

DNA was extracted with QIAamp DNA Blood Mini Kit (Qiagen) according to the manufacturer’s instructions (protocol “DNA Purification from Blood or Body Fluids” in QIAamp DNA Mini and Blood Mini Handbook 05/2016) with some modifications. As starting material for the extraction 25 µl serum and 75 µl PBS were used, volumes of protease, buffer AL and ethanol were 10 µl, 100 µl and 100 µl, respectively, and the DNA was eluted in 60 µl of water. To measure mtDNA and nDNA in the extracted DNA ddPCR methodology (BioRad) was used. This is a sensitive method based on a technology where thousands of droplets of a water-oil emulsion are created for each sample and a PCR reaction is performed in each individual droplet. The result is given as absolute concentration in the sample and very low quantities can be detected. In each reaction two sequences can be amplified at the same time, in this study mtDNA and nDNA. A reaction mix was prepared by adding for each reaction 11 µl supermix (ddPCR SMX for Probes, no dUTP, cat no 1863023, BioRad), 1.1 µl mtDNA primer (ddPCR GEX Assay MT-DN1 Hsa FAM, dHsaCPE5029120, cat no 10031252, BioRad), 1.1 µl nDNA primer (ddPCR CNV Assay EIF2C1 Hsa HEX, dHsaCP1000002, cat no 10031243, BioRad) and 1 µl Hind III restriction enzyme (5 U/reaction, recombinant 10.000 units, cat no R0104S, BioNordika), and then 14.2 µl of the mix was added to 7.8 µl of extracted DNA. After incubation at RT for 20 min, to enable Hind III to cleave the DNA into smaller fragments, droplets were generated by inserting the samples into an automatic droplet generator (QX200 Droplet Generator, BioRad) followed by PCR amplification in a thermal cycler (C1000 Touch Thermal Cycler, BioRad). To stabilize the droplets the amplified product was incubated over night at +4°C. Then the plate was inserted into a droplet reader (QX200 Droplet Reader, BioRad) and the results were presented as copies/µl in the QuantaSoft software (version 1.7.4, BioRad). A positive control and a no template control were included in each run. A manual cut-off for positive droplets was set for mtNDA and nDNA that worked well for all samples. Samples with less than 10 000 droplets in total were rerun and samples with positive droplets only were diluted and rerun.

#### C-Peptide, HLA Typing, Autoantibody and Cytokine Assays

Methods for c-peptide, HLA typing and autoantibody measurements have been described previously (27). Briefly, levels of GADA, IA-2A and ZnT8A (RA-, WA- and QA-variants) were determined by radiobinding assays and IAA was measured with a competitive radiobinding assay. Results were expressed as Units/ml in relation to a standard curve. HLA genotyping was performed on HLA class II regions DQB1, DQA1 and DRB1, and genotypes were classified into the risk categories low, moderate and high. C-peptide was measured with a time-resolved fluoroimmunoassay. IL-1beta was measured with an IL-1beta human ELISA kit (Invitrogen) and IFN was detected with a Luminex assay using Bio-Plex Pro Cytokine Panel (Bio-Rad), as previously described (26).

### Statistics

Statistical analyses were performed with IBM SPSS Statistics (version 24) and GraphPad Prism (version 8.3.0). Due to nonparametric data Mann-Whitney test was used for comparison between groups and Spearman test for correlations. Friedman’s test followed by Dunn’s multiple comparisons test was used for analyses over time within the group. For comparisons over time within the high-risk group Kruskal-Wallis test followed by Dunn’s multiple comparisons test was used instead of Friedman’s test due to the presence of missing data at all time points. A p value of < 0.05 was considered significant.

## Results

### Increased Levels of NETs After T1D Onset

**Figure 1:**
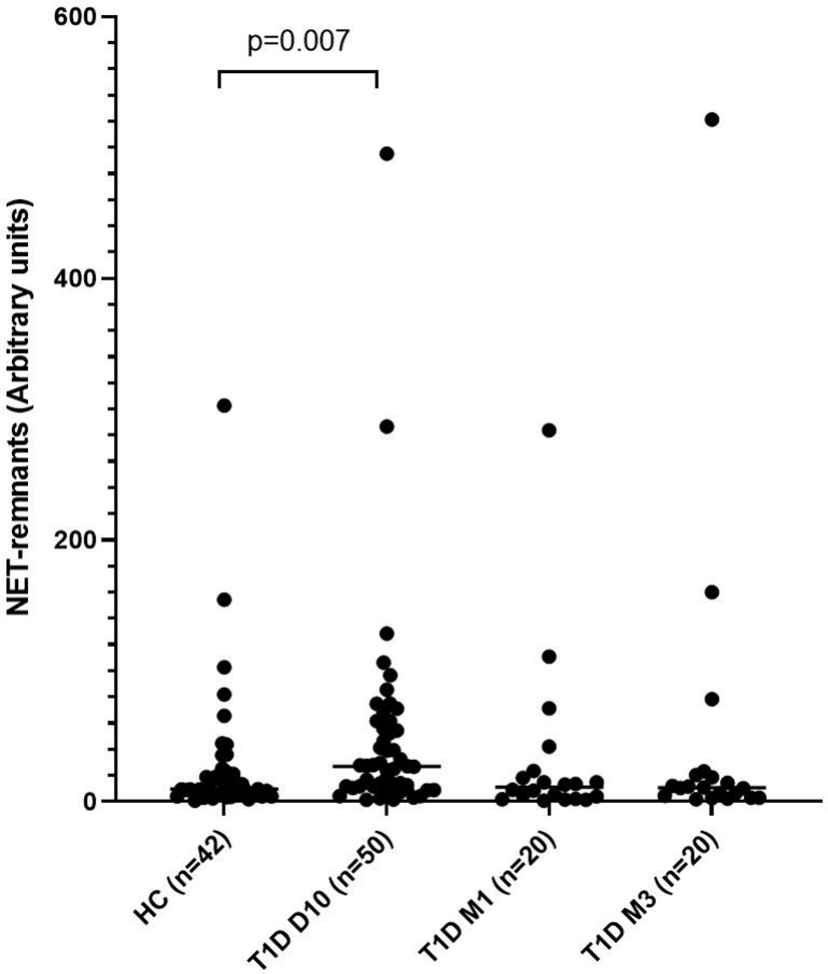
Levels of NETs in children with newly diagnosed type 1 diabetes (T1D) and healthy children (HC). Healthy children (n=42) were compared with children after onset of T1D; 10 days (n=50), 1 month (n=20) and 3 months (n=20) after onset (cohort 1, Mann-Whitney test).

**Figure 2:**
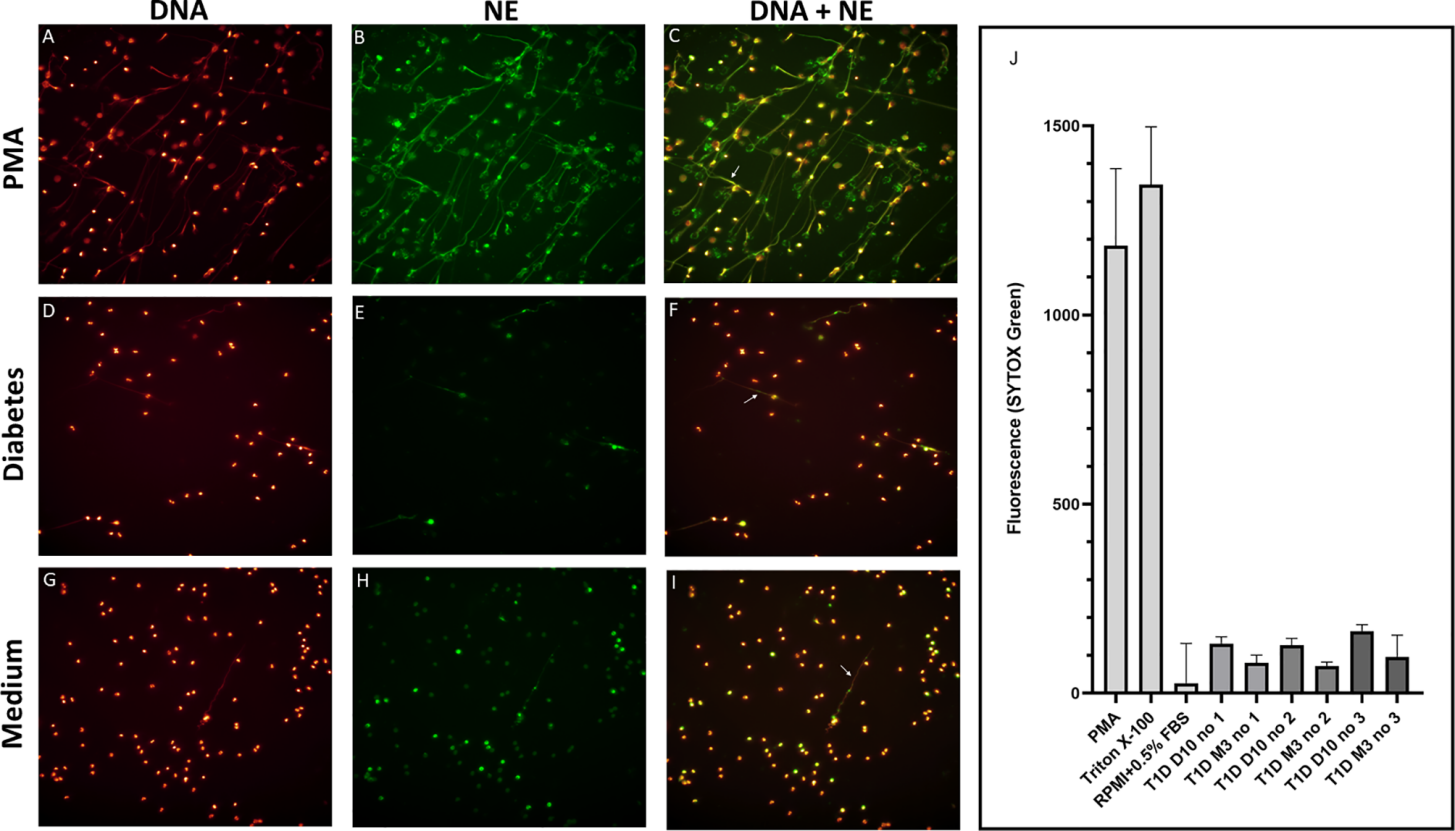
Visualization and quantification of NETs. Neutrophils were isolated and cultured for 4h at 37°C with or without 20 nM PMA or with 5% serum from T1D children. NETs were visualized by immunofluorescence microscopy using a 25× objective and defined as extracellular co-localisation of DNA and neutrophil elastase (NE). DNA was labelled with DAPI (glow, red) and NE with an Alexa Fluor 488–conjugated antibody (green). Neutrophils from a healthy control were either stimulated with **(A–C)** PMA, **(D–F)** serum from a diabetes patient (day 10 in the illustration) or **(G–I)** left unstimulated. Arrows in the merged images indicate NETs **(C, F, I)**. **(A–I)** in this figure is not intended for quantification of NETs but selected to demonstrate that neutrophils can produce NETs when stimulated with serum from diabetes patients. Quantification of NETs using Sytox Green is shown in **(J)**. PMA, Triton X-100 and medium (RPMI + 0.5% FBS) were used as controls. Serum from three T1D patients 10 days and 3 months after diagnosis were used to induce NETs. NETs, neutrophil extracellular traps; PMA, phorbol-12-myristate-13-acetate; FBS, fetal bovine serum.

Levels of NETs were significantly increased (185% increase, p=0.007) in newly diagnosed T1D children (n=50) 10 days after disease onset compared to healthy individuals (n=42) (cohort 1, **Figure 1**), measured by the NET-remnants ELISA detecting MPO-DNA complexes. These NET-levels correlated with levels of neutrophil elastase in the serum samples (r=0.496, p=0.002, n=36). The *in vitro* assays for generation of NETs showed that serum from T1D children in cohort 1 (n=3) stimulated neutrophils from a healthy individual to produce NETs (**Figure 2**). Serum 10 days after disease onset induced more NETs compared to serum 3 months after onset (10% and 6% of positive control, respectively). *In vitro* degradation of NETs was similar with serum from T1D children (n=3) as from healthy children (n=3) (**Supplementary Figure 2**).

### Increased Levels of mtDNA and nDNA After T1D Onset

**Figure 3:**
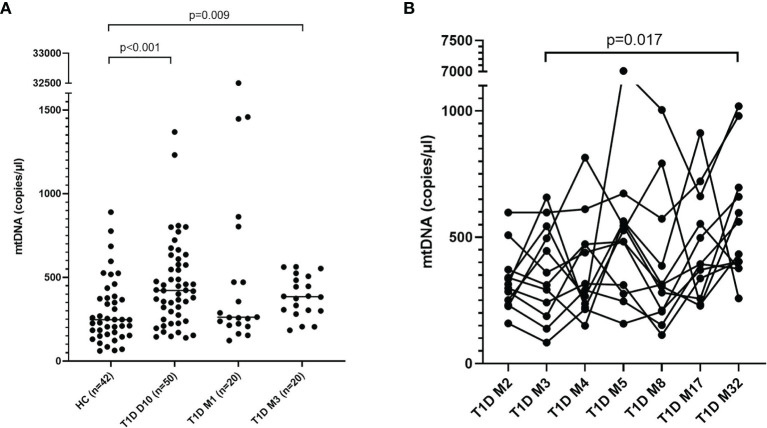
Levels of mtDNA in children with newly diagnosed type 1 diabetes (T1D). Healthy children (n=42) compared with children after onset of T1D; 10 days (n=50), 1 month (n=20) and 3 months (n=20) after onset (cohort 1, Mann-Whitney test) **(A)**. Levels of mtDNA over time in paired samples from T1D children (n=12) in cohort 2 (Friedman’s test followed by Dunn’s multiple comparisons test) **(B)**.

**Figure 4:**
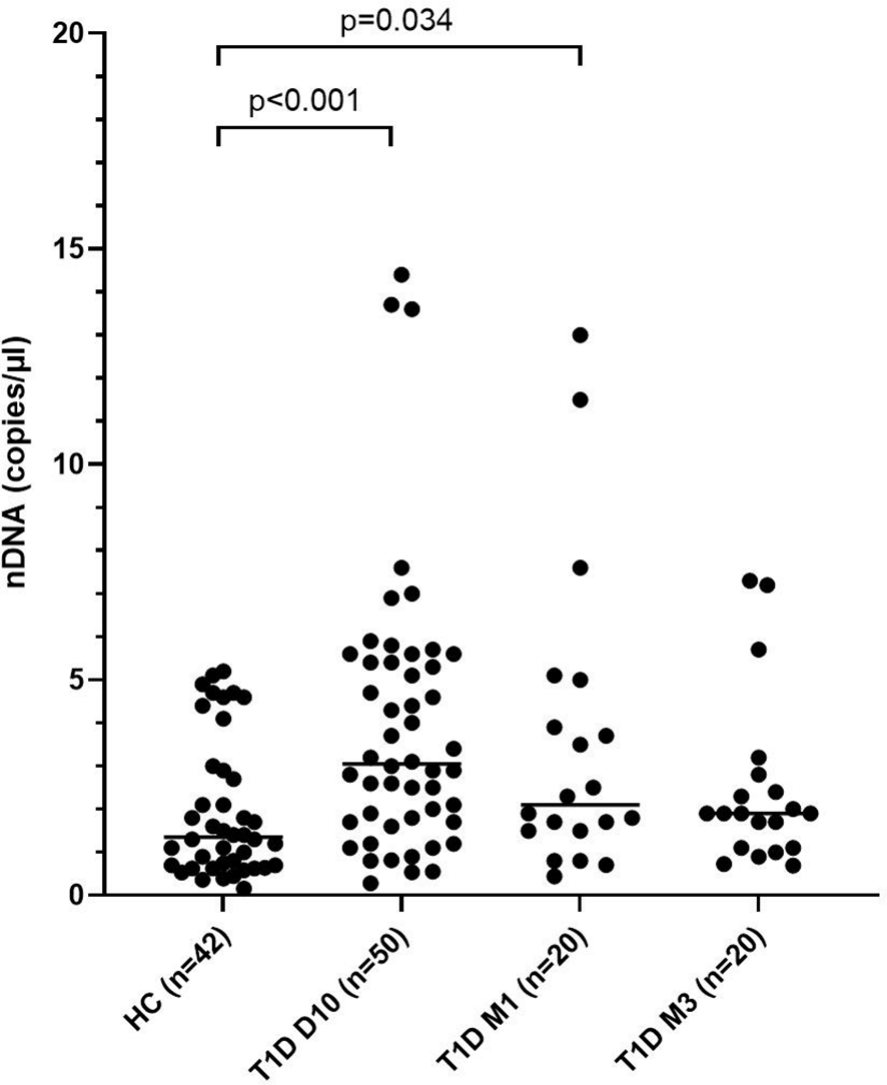
Levels of nDNA in children with newly diagnosed type 1 diabetes (T1D) and healthy children (HC). Healthy children (n=42) were compared with children after onset of T1D; 10 days (n=50), 1 month (n=20) and 3 months (n=20) after onset (cohort 1, Mann-Whitney test).

Levels of mtNDA were significantly higher (71% increase, p<0.001) in newly diagnosed T1D children (n=50) 10 days after disease onset (cohort 1) compared to healthy children (**Figure 3A**). Samples from 20 children of the T1D group were also collected at 1 and 3 months (**Supplementary Figure 1**). In these samples, mtDNA was elevated at 3 months (56% increase, p=0.009, **Figure 3A**). Analysis of extracellular DNA levels in serial samples from another group of newly diagnosed T1D children (n=12, cohort 2) showed that mtDNA was increased 32 months after disease onset (48% increase, p=0.017, **Figure 3B**). In the 50 newly diagnosed T1D children 10 days after disease onset (cohort 1) levels of nDNA were increased (126% increase, p<0.001) compared to healthy children (**Figure 4**). In samples from the T1D group that were collected also at 1 and 3 months (n=20, cohort 1), nDNA was increased at 1 month (56% increase, p=0.034) compared to healthy children.

### Fluctuating Levels of Extracellular DNA in Children With High Risk of T1D

**Figure 5:**
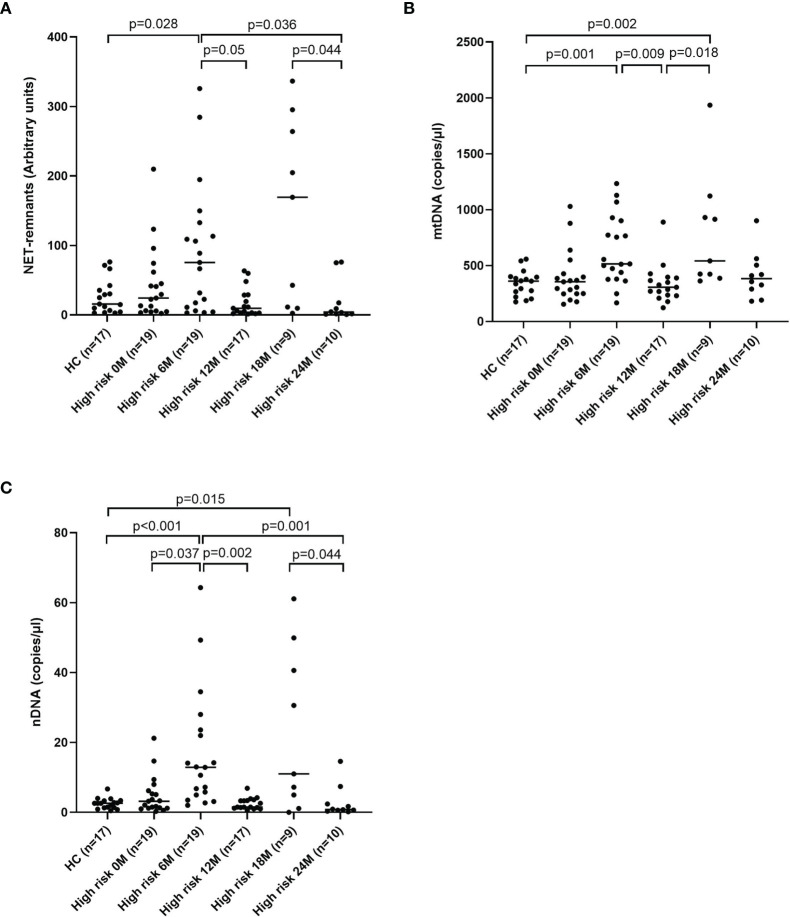
Healthy children compared with children at high risk of T1D at five time points for NETs **(A)**, mtDNA **(B)** and nDNA **(C)** (Mann-Whitney test). Comparison over time within the high-risk group was done using Kruskal-Wallis test followed by Dunn’s multiple comparisons test **(A–C)**.

**Figure 6:**
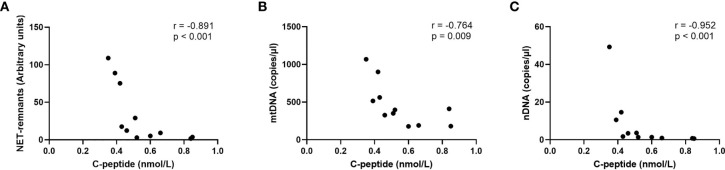
Correlations for NETs **(A)**, mtDNA **(B)** and nDNA **(C)** with c-peptide in the last sample before onset in children who progressed to T1D (Spearman correlation).

Levels of extracellular DNA were similar in the baseline samples from children with high risk of T1D (cohort 3) as in healthy children (**Figures 5A–C**). Analysis of samples collected during the follow-up showed that levels of NETs increased at 6 months, and that mtDNA and nDNA increased at 6 and 18 months. However, at 12 months and 24 months levels of extracellular DNA were not increased compared to healthy children. Paired analysis of samples over time showed that levels from the same individual fluctuated over time with peaks at 6 and 18 months (**Figures 5A–C**). The fluctuating levels of extracellular DNA were not related to the month the sample was taken (data not shown). Stratification of samples from the high-risk group into progressors (n=11) and non-progressors to T1D (n=9) did not show any differences of the extracellular DNA levels at any time point (data not shown). Comparison of the samples from progressors with healthy controls revealed increased NETs, mtDNA and nDNA at 6 months (p=0.041, p=0.015, p<0.001, respectively) and increased mtDNA at 18 months (p=0.004). Since the time points for sample collection were not related to the time until diabetes onset, the last sample before onset for each progressor was assembled in one group and analysed separately (**Supplementary Table 1**). Levels of extracellular DNA in the last sample was compared with healthy controls and with non-progressors, showing no significant differences. However, in the last sample there were strong negative correlations with c-peptide for NETs, mtDNA and nDNA (**Figure 6**, r=-0.891, p<0.001; r=-0.764, p=0.009; r=-0.952, p<0.001, respectively).

### Correlations Between NETs, mtDNA and nDNA

The relationship between NETs, mtDNA and nDNA was studied, both for all samples together (cohort 1-3) and stratified into healthy controls (cohort 1 and cohort 3 separately), T1D and high-risk individuals. NETs correlated strongly with mtDNA and nDNA, and mtDNA correlated with nDNA, both when samples were analysed together and when stratified in the subgroups (**Table 2**). Only in healthy controls from cohort 3 correlations between mtDNA and NETs, as well as between mtDNA and nDNA, were not significant.

**Table 2:** Correlations between NETs, mtDNA and nDNA for all samples analysed in this study (cohort 1-3) at all time points and for T1D, healthy controls in cohort 1 (HC1), healthy controls in cohort 3 (HC3) and high-risk individuals (HR) separately.

Spearman		All samples (n=314)		T1D (n=178)		HC1 (n=42)		HC2 (n=17)		HR (n=77)	
correlation		mtDNA	nDNA	mtDNA	nDNA	mtDNA	nDNA	mtDNA	nDNA	mtDNA	nDNA
NET-remnants	r	0.383	0.775	0.410	0.757	0.402	0.679	0.181	0.912	0.423	0.844
	p	<0.001	<0.001	<0.001	<0.001	0.008	<0.001	ns	<0.001	<0.001	<0.001
mtDNA	r		0.541		0.567		0.706		0.113		0.540
	p		<0.001		<0.001		<0.001		ns		<0.001

ns, non-significant.

### Extracellular DNA in Relation to Age and Sex

**Figure 7:**
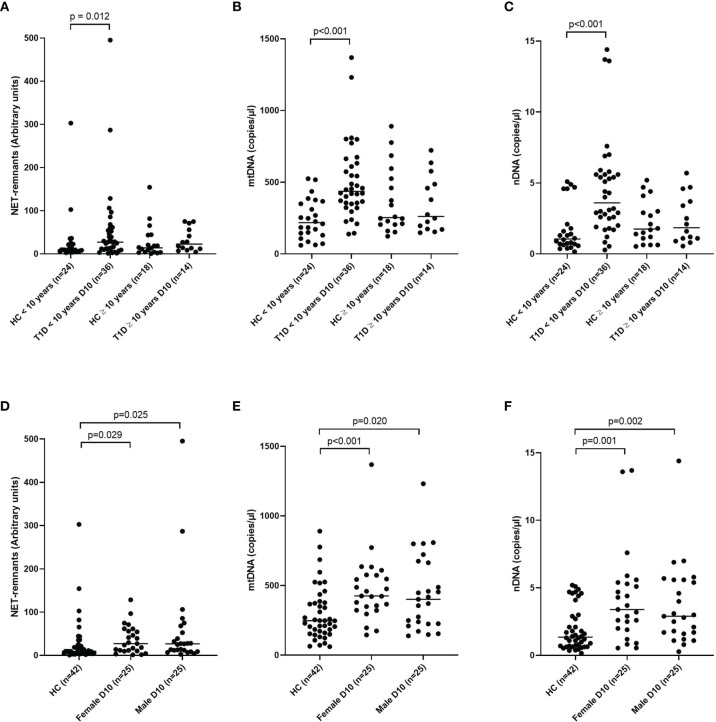
T1D and healthy children in cohort 1 subgrouped into younger than 10 years and 10 years or older for NETs **(A)**, mtDNA **(B)** and nDNA **(C)** and subgrouped into male and female for NETs **(D)**, mtDNA **(E)** and nDNA **(F)** (Mann-Whitney test). Gender in the healthy control group was not available. HC, healthy control.

NETs and nDNA correlated negatively with age when all samples (cohort 1-3) at all time points were grouped in the analysis (r=-0.174, p=0.002 and r=-0.198, p<0.001, respectively, **Supplementary Table 2**). Comparison by group definition showed that the T1D group was responsible for the correlation with age (NETs r=-0.210, p=0.005 and nDNA r=-0.299, p<0.001, respectively). Classification of both T1D and healthy controls (cohort 1) into children younger than 10 years and children 10 years or older showed that T1D children younger than 10 years had increased levels of extracellular DNA compared to healthy children with the same age, while no difference was observed between T1D and healthy controls ≥ 10 years old (**Figures 7A–C**). Extracellular DNA did not correlate with sex or weight neither in all samples nor in the subgroups (T1D, high-risk and healthy controls), except for nDNA and sex in the T1D group (r=0.188, p=0.012, **Supplementary Table 2**). When the T1D group in cohort 1 was divided according to sex, both female and male individuals showed increased levels of extracellular DNA compared to healthy children (**Figures 7D–F**).

### C-Peptide and Blood Glucose in T1D Children

A weak negative association was observed in T1D samples 10 days after onset between HbA1c and NETs, mtDNA and nDNA (r=-0.393, p=0.012; r=-0.396, p=0.012; r=-0.368, p=0.019, respectively). Fasting blood glucose at 10 days, 1 month and 3 months as well as blood glucose at 120 min of OGTT measured at 3 months did not correlate with extracellular DNA (data not shown). To investigate if levels of extracellular DNA in newly diagnosed T1D samples were related to the rate of loss of endogenous insulin production, the T1D children in cohort 1 were stratified into those with early loss of insulin production (n=20, defined as undetected c-peptide levels <0.02 nmol/L within 2 years after diagnosis) or late loss (n=20, remaining endogenous insulin production 4 years after onset), while the children with intermediate loss of insulin production (n=10) were excluded from this analysis (**Table 3**). Extracellular DNA levels did not differ between the two groups in samples 10 days after disease onset. However, c-peptide and age were lower (p<0.001 and p=0.005, respectively) and f-glucose was higher (p=0.008) in the group that lost insulin production early, while HbA1c did not differ between the groups (**Table 3**). Since increased levels of extracellular DNA was found primarily in T1D children below the age of 10 (**Figures 7A–C**), levels of c-peptide, HbA1c and blood glucose were compared between children younger than 10 years and children 10 years or older. Levels of c-peptide (median value 0.08 and 0.14, respectively, p=0.006) and HbA1c (median value 99 and 143 mmol/mol, respectively, p=0.007) were lower in T1D children below 10 years of age. HbA1c correlated negatively with levels of NETs 10 days after disease onset in the younger children (r=-0.428, p=0.023). In addition, the T1D children below 10 years of age had more often an early loss of insulin production compared to older children (n=18 and n=2, respectively, p=0.002). There were no differences in levels of extracellular DNA in the younger children with early loss compared to late loss of endogenous insulin production.

**Table 3:** Characteristics of T1D children with early (< 2 years) and late (> 4 years) loss of endogenous insulin production.

	Early (< 2 years)	Late (> 4 years)	p-value
Definition of group	Loss of insulin production	Remaining insulin production	
	within 2 years after onset	4 years after onset	
Individuals [number, (male/female)]	20 (8/12)	20 (11/9)	
Age at onset [years, IQR)]	6.8 (6.0-9.1)	10.8 (8.5-11.9)	0.005
C-peptide at 10 days [nmol/L, (IQR)]	0.07 (0.01-0.11)	0.14 (0.11-0.23)	<0.001
Fasting glucose at 0-10 days [mmol/L, (IQR)]	10.9 (6.9-15.7)	5.2 (3.9-6.5)	0.008
HbA1c at 0-10 days [%, (IQR)]	12.8 (9.9-66.3)	12.9 (9.5-30.9)	ns
NET-remnants at 10 days (arbitrary units)	20.7 (8.9-59.8)	26.9 (12.2-53.6)	ns
mtDNA at 10 days (copies/µl)	440 (328-618)	373 (180-527)	ns
nDNA at 10 days (copies/µl)	3.0 (1.7-5.8)	2.6 (1.1-3.9)	ns

Endogenous insulin production was measured as c-peptide levels in samples 10 days after onset of the disease. ns, non-significant.

### Cytokines, HLA and Autoantibodies in T1D Children

Measurement of IL-1beta in serum from T1D and healthy children resulted in very low levels. Only 14 of the samples in cohort 1 had detectable levels (7 from T1D and 7 from healthy controls) and one of the T1D samples was within the range of the standard curve (3.9-250 pg/mL). IFN levels were similar over time in samples from T1D children in cohort 2, with a median value of 0.49-0.57 pg/ml for the first five visits (samples from the last two visits were missing), and did not correlate with mtDNA (data not shown).

In samples from all T1D children (cohort 1) as well as from children below 10 years of age at day 10 nDNA correlated with positivity for ZnT8RA(Arg) (r=0.670, p=0.024, n=11 and r=0.822, p=0.007, n=9, respectively) and with positivity for any of the three ZnT8A antibody variants (Arg, Glt, Trp) (r=0.837, p=0.001, n=11 and r=0.822, p=0.007, n=9, respectively). No association between HLA-risk and extracellular DNA was found (data not shown).

## Discussion

In this study, we found increased levels of NETs and mtDNA in newly diagnosed T1D children short after disease onset. The increased levels of NETs are in agreement with a previous study (4), however, in our study the T1D children were younger (median age 8 vs 15 years in Wang’s study). It was interesting that higher extracellular DNA levels were observed short after onset. Unexpectedly, this increase was only found in the younger children, which might reflect the more rapid course of beta cell destruction that often occurs in children at lower ages (31). This idea is supported by our results showing lower c-peptide levels and a more rapid loss of beta cell function in the group of children younger than 10 years of age. The lack of association of extracellular DNA and age in the healthy children was in agreement with one study showing that mtDNA increased with age only in individuals above 50 years of age, whereas children and adults younger than 50 years had similar levels (32). Previous studies have measured mtDNA in peripheral blood, i.e. intra- and extracellular mtDNA together, in adults with T1D, type 2 diabetes (T2D) and pre-diabetes individuals (33–35). Recently, increased extracellular mtDNA levels in serum from adult T1D patients were found (25). To our knowledge, this is the first study showing increased levels of mtDNA in serum from T1D children short after disease onset.

Increased levels of extracellular DNA in newly diagnosed T1D children might reflect the inflammatory process in the pancreas and predict future loss of insulin production. However, in our study we found no differences in levels of extracellular DNA between patients with rapid and slow loss of endogenous insulin production. Instead, lower age, lower c-peptide and higher fasting glucose 10 days after diagnosis were found in children who lost their insulin production early, which has been previously observed in newly diagnosed T1D patients (36–40). In our study, lower c-peptide levels associated with higher extracellular DNA in the last sample before onset in high-risk children.

The increased nDNA observed in T1D children short after disease onset suggests increased cell death. The strong correlations in our study between NETs, mtDNA and nDNA support the idea that the cells mainly have died through NETosis and that the formed NETs contain both nDNA and mtDNA. The increased mtDNA 10 days after disease onset in our study was probably due to enhanced NETosis. MtDNA can also be actively secreted from living cells during vital NETosis (9) or *via* interferogenic DNA webs (10). The observed increase in mtDNA over time in consecutive samples was not accompanied by increased nDNA and it was thus more likely actively released by living neutrophils into the blood. Both extracellular mtDNA and NETs are inflammatory mediators and can exert their function both indirectly by activation of immune cells to produce inflammatory cytokines or directly by causing cell damage (7, 12, 41). MtDNA can induce inflammation by activation of toll-like receptor 9 (TLR9), inflammasomes, and as a stimulator of interferon genes (STING) pathway (42). It has been shown that increased extracellular mtDNA in serum from T1D patients was accompanied with caspase-1 and IL-1beta activation, indicating NLRP3 inflammasome activation (25). In patients with type 2 diabetes, increased mtDNA levels were associated with IL-1beta levels and may contribute to chronic melanoma 2 (AIM2) inflammasome mediated inflammation (43). IL-1beta was measured in the samples included in our study, but since only a few samples had detectable levels in serum, it was not possible to draw any conclusions from the analysis. In addition, we found no correlation between mtDNA and IFN levels in T1D samples over time.

Increased NETs in the circulation could be caused either by increased production of NETs and/or reduced capacity to degrade the NETs. Our results that T1D serum *in vitro* induced NETs on neutrophils from a healthy individual and that serum 10 days after diagnosis induced more NETs compared to 3 months after diagnosis indicates that the production of NETs was increased short after diagnosis, although further experiments are needed to verify this finding. In one study, the DNase activity in serum was shown to be lower in T1D patients compared to healthy controls (44). Our NET degradation experiment did not show any clear difference in degradation between T1D children and healthy controls, but more individuals need to be analysed to clarify if the capacity to degrade NETs is reduced in T1D. Other possible explanations, besides inflammation, for increased levels of NETs and mtDNA in T1D might be high blood glucose levels, and autoantibody titres and numbers. It has been shown that neutrophils under hyperglycaemic conditions produce more superoxide and cytokines (45), which could result in hyperglycaemia-induced ROS mediated mtDNA damage and release of mtDNA to the circulation (35). In addition, high glucose induces NETosis *in vitro* and in patients with T2D (21, 46). In contrast, high glucose concentrations were found to decrease formation of NETs in two other studies (47, 48). This is in line with our results, showing negative correlations for both NETs and mtDNA with HbA1c in T1D children short after disease onset, suggesting that the increased levels of extracellular DNA are not a consequence of high blood glucose. The number of positive autoantibodies in T1D patients has been associated with levels of neutrophil elastase and proteinase 3, both associated with NETs (4), with higher levels in the T1D patients positive for higher number of autoantibodies. We could, however, not find any association for NETs and mtDNA with autoantibodies. There was a correlation for nDNA and ZnT8A, but few samples were included in the analysis, making it difficult to interpret.

To test our hypothesis that children with high risk of T1D might have higher levels of extracellular DNA we studied a group of healthy children with multiple autoantibodies at five time points. The results revealed fluctuating levels, sometimes increased compared to healthy autoantibody negative children. The reason for this variation is not clear. The fluctuating levels of extracellular DNA were not related to progress to T1D, to the month the sample was taken or to clinical parameters such as c-peptide or HbA1c. However, similar fluctuation of NETs, mtDNA and nDNA suggests increased cell death at some time points but not at others. It might be possible that these variations reflect a fluctuating autoimmune process at certain time points, e.g. during infections or due to other stress factors.

The extracellular traps measured with the ELISA detecting MPO-DNA complexes are probably not solely originating from neutrophils, but there might also be extracellular traps released from monocytes, since monocytes also express MPO and it has been shown that they can release extracellular traps as well (49). Our finding that neutrophil elastase in the samples correlated with extracellular traps measured with the MPO-DNA complex ELISA, suggests that neutrophil elastase, which is considered specific for neutrophils, was present in the extracellular traps that we measured and thus mainly were of neutrophil origin. Another factor that might influence the levels of extracellular DNA is storage temperature of the samples (50). In our study, storage temperature differed between the samples, but by having different cohorts of samples with for example two healthy control groups only samples stored at the same temperature were used for comparison, making it unlikely that the storage temperature influenced the results.

In summary, we found increased levels of extracellular DNA in newly diagnosed T1D children, but levels of extracellular DNA shortly after onset do not reflect future loss of endogenous insulin production. Higher levels of nDNA in T1D individuals short after disease onset suggest increased cell death, and the increased NETs and mtDNA might be explained by an ongoing inflammation. Extracellular DNA does not seem to be a suitable biomarker candidate for prediction of T1D, at least not in high-risk children positive for multiple autoantibodies.

## Data Availability Statement

The raw data supporting the conclusions of this article will be made available by the authors, without undue reservation.

## Ethics Statement

The studies involving human participants were reviewed and approved by The Research Ethics Committee of the Faculty of Medicine and Health Sciences at Linköping University, the Medical Faculty at Lund University and the Medical Product Agency in Sweden. Written informed consent to participate in this study was provided by the participants’ legal guardian/next of kin.

## Author Contributions

CS, JL, DA and RC designed the study. DA and CS set up methodology. CS and IJ conducted the experiments. CS analysed the data and wrote the manuscript. CS, DA, IJ, RC and JL revised the manuscript. All authors contributed to the article and approved the submitted version.

## Funding

This research was supported by the Swedish Child Diabetes Foundation (Barndiabetesfonden, http://www.barndiabetesfonden.se) and the Ingrid Asps Foundation. ABIS was also supported by Swedish Research Council (K2005-72X-11242-11A and K2008-69X-20826-01-4), JDRF Wallenberg Foundation (K 98-99D-12813-01A), Medical Research Council of Southeast Sweden (FORSS) and Östgöta Brandstodsbolag.

## Conflict of Interest

The authors declare that the research was conducted in the absence of any commercial or financial relationships that could be construed as a potential conflict of interest.

## Supplementary Material

Supplementary Figure 1 Summary of the study population including cohort number, status of the individual (healthy, T1D, high risk), number of individuals in the group, median age at first sampling, time points for serum sample collection and storage temperature for the samples. D10 = 10 days after diagnosis, MX = X months after diagnosis, for high-risk individuals XM is X months after baseline sample.

Supplementary Figure 2 Quantification of NET degradation using PicoGreen. PMA-induced NETs were incubated with 1% serum from three T1D patients 10 days after diagnosis (D10) and three healthy controls for 16h at 37°C. HC = healthy control; T1D = type 1 diabetes.

Supplementary Table 1 Characteristics of the last sample before onset in progressors compared with healthy controls.

Supplementary Table 2 Correlations between extracellular DNA and age, sex and weight for all samples analysed in this study (cohort 1-3) at all time points and for T1D, healthy controls (HC) and high-risk individuals (HR) separately. Numbers within brackets indicate number of samples where age, sex and/or weight were available.
