# Supplementary figures and images for: Increase of Neutrophil Extracellular Traps, Mitochondrial DNA and Nuclear DNA in Newly Diagnosed Type 1 Diabetes Children but Not in High-Risk Children
Source: Front Immunol. 2021 Jun 15;12:628564. doi: 10.3389/fimmu.2021.628564 (PMC8239297; doi:10.3389/fimmu.2021.628564)

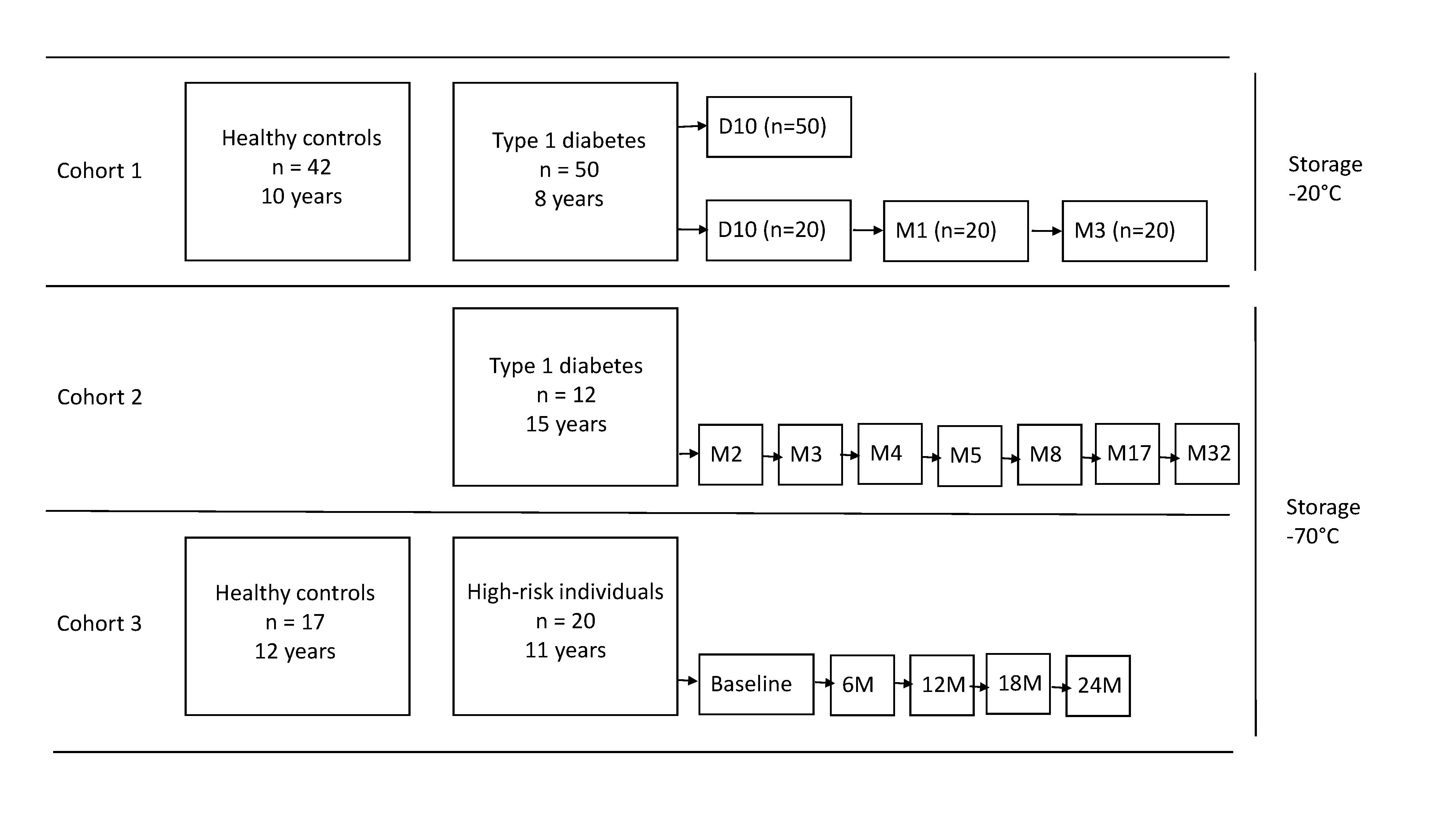

Supplement: Supplementary Figure 1 — Summary of the study population including cohort number, status of the individual (healthy, T1D, high risk), number of individuals in the group, median age at first sampling, time points for serum sample collection and storage temperature for the samples. D10 = 10 days after diagnosis, MX = X months after diagnosis, for high-risk individuals XM is X months after baseline sample. [file Image_1.jpeg]

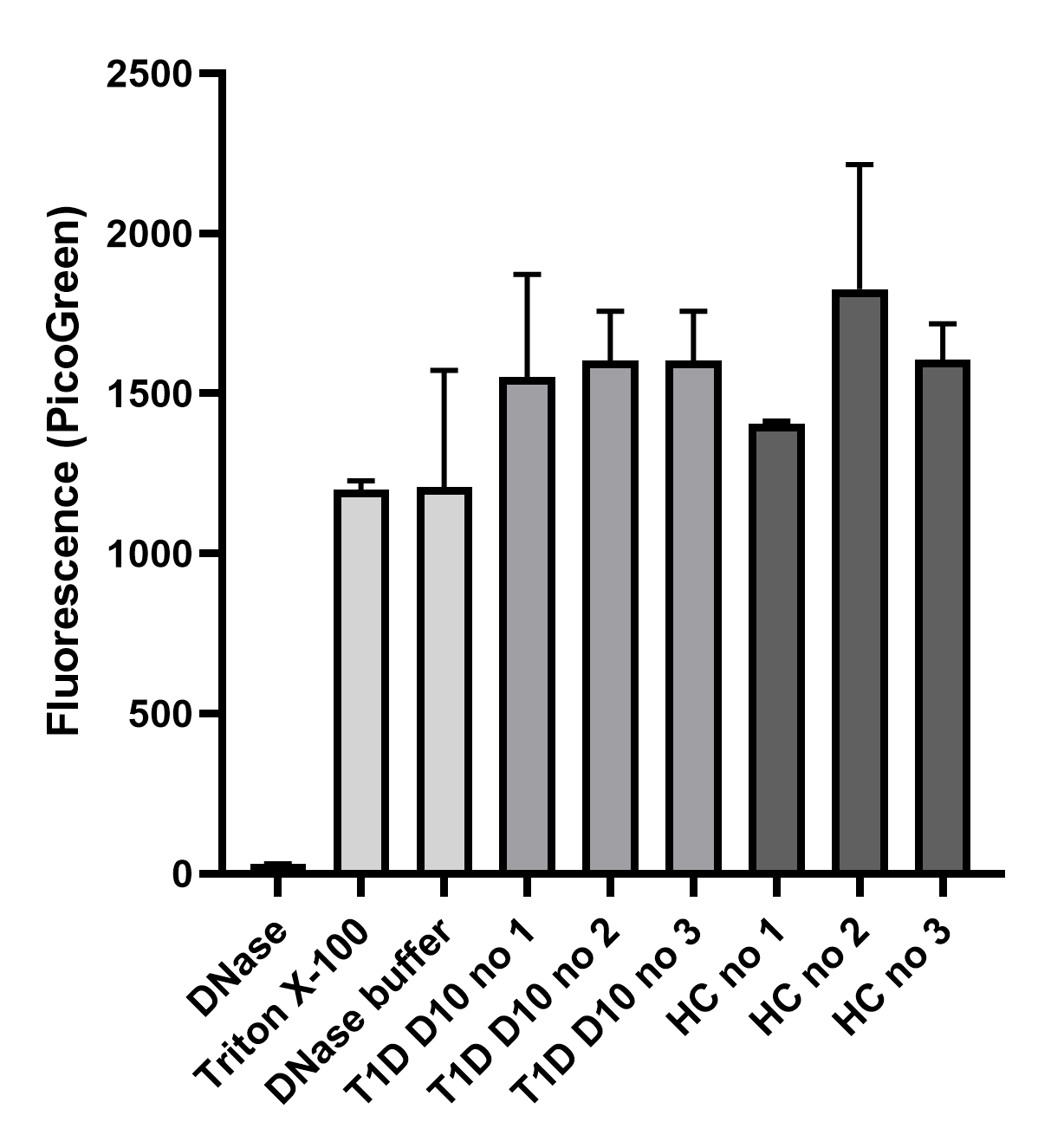

Supplement: Supplementary Figure 2 — Quantification of NET degradation using PicoGreen. PMA-induced NETs were incubated with 1% serum from three T1D patients 10 days after diagnosis (D10) and three healthy controls for 16h at 37°C. HC = healthy control; T1D = type 1 diabetes. [file Image_2.tif]
